# Strategies for Minimizing Environmental Impact in Construction: A Case Study of a Cementitious 3D Printed Lost Formwork for a Staircase †

**DOI:** 10.3390/ma18040825

**Published:** 2025-02-13

**Authors:** Sophie Viktoria Albrecht, Stefan Hellerbrand, Florian Weininger, Charlotte Thiel

**Affiliations:** Department of Construction Materials, OTH Regensburg, 93053 Regensburg, Germanycharlotte.thiel@oth-regensburg.de (C.T.)

**Keywords:** additive manufacturing in construction (AMC), life cycle assessment (LCA), 3D concrete printing (3DCP), selective cement activation (SCA), permanent formwork

## Abstract

The construction industry faces significant challenges, including environmental sustainability, rising material costs, and a shortage of skilled labor. Digital fabrication technologies offer innovative solutions to address these issues by reducing raw material consumption and waste generation. Among these, 3D printing technologies offer distinct advantages over traditional construction methods, particularly in handling complex geometries. However, the significant environmental impact of cement in 3D printed concrete, due to its high rheological and printability requirements, remains a concern. This study introduces a novel application of 3D printed permanent formwork in the construction of a winder staircase, assessed through an Environmental Life Cycle Assessment (LCA) from cradle to gate. By comparing the environmental impacts of various construction materials and processes, this study highlights the comparative advantages and disadvantages of conventional methods versus 3D printing. The LCA results reveal that traditional production methods, particularly those using plywood formwork, exhibit higher environmental impacts. In contrast, timber formwork performs better than most 3D printed mixtures in terms of Global Warming Potential (GWP), Acidification Potential (AP), and abiotic depletion potential (ADP). The findings of this study underscore the potential of additive manufacturing for sustainable construction, particularly through the use of low-clinker cement in 3D printed formwork, offering a promising pathway towards reducing the environmental footprint of construction activities.

## 1. Introduction

The modern construction industry has to tackle the shortage of skilled workers [1], the reduction in negative environmental impact [2] and the housing shortage [3]. Sustainability and resource efficiency are crucial for the construction industry, which is one of the world’s largest consumers of natural resources and energy [2]. The construction sector is responsible for a significant proportion of global carbon dioxide emissions, both through the production of building materials such as concrete and steel, and through the energy consumed during the construction and use of buildings [4]. In the face of climate change and resource scarcity, it is essential that the construction industry develops and implements more sustainable and resource-efficient practices. Through the use of efficient technologies, waste reduction and the conscious use of materials, the construction industry can significantly reduce its environmental footprint while ensuring the long-term availability of resources [5].

Comprehensive assessments like Life Cycle Analysis (LCA) are becoming increasingly important to assess and reduce potential environmental impacts [6,7].

An Environmental Life Cycle Assessment (E-LCA) is a method used to evaluate the environmental impacts associated with all stages of a product’s life, from raw material extraction to disposal. In addition, even supplementary information beyond the building’s life cycle can be considered as foreseen in EN 15804 phase D [8]. This provides a full picture of the product’s environmental footprint, including emissions and waste produced along the way.

Recent advancements, such as additive manufacturing in construction, commonly known as 3D printing, offer potential solutions to the aforementioned challenges. The study by Garcia de Soto et al. demonstrates that additive manufacturing in construction has the potential to significantly enhance productivity in the fabrication of complex structures [9].

Agustí-Juan et al. [10] investigated the environmental impact of a digital fabrication method compared to traditional construction techniques for complex architectural forms. The LCA comparison between digital fabrication and conventional construction methods for various wall types shows that conventional methods have lower environmental impacts for simpler walls, but as structural complexity increases, the environmental performance of conventional walls deteriorates. For double-curved and complex walls, Mesh Mold offers significant reductions in climate change (38%) and human toxicity (31%) compared to conventional techniques using polystyrene formwork. In addition, the environmental impact of conventional methods increases with form complexity, whereas the impact of the Mesh Mold process remains constant, making it more advantageous for complex architectural designs [10].

Conventional one-time use wood formwork, particularly when utilizing plywood, exhibits significant potential for improvement in terms of environmental impact compared to plastic or steel formwork [11,12]. To investigate these aspects, this study utilizes a case study of a staircase, comparing conventional formwork with 3D printed permanent formwork, while also analyzing various databases to assess the impact of cement and researching different electricity mixes to evaluate their influence on the overall environmental performance. Through this comparison, the study aims to evaluate the potential of additive manufacturing in reducing the environmental impact of construction activities.

## 2. Materials and Methods

### 2.1. Goal and Scope

**Figure 1:**
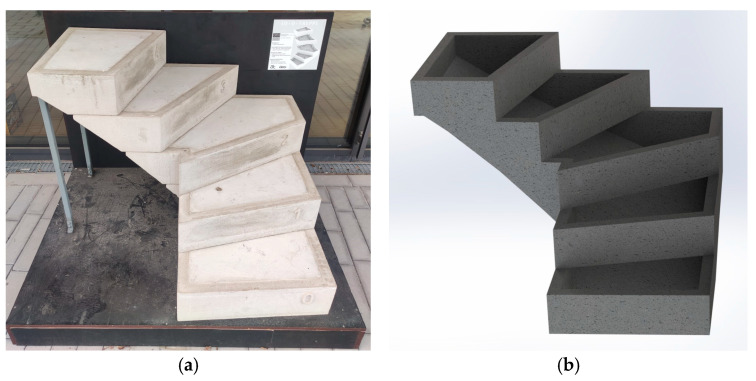
A case study of a 3D printed lost formwork for a winding staircase, demonstrating material efficiency and adaptability: (**a**) the produced additively manufactured lost formwork staircase filled with concrete, serving as the final exhibition piece; (**b**) a rendering of the additively manufactured lost formwork staircase.

For the Life Cycle Assessment (LCA), the framework was developed in ISO 14040 and ISO 14044 was applied [13,14]. The software openLCA 2.2 and the database ecoinvent3.9.1 cut-off were used. The life cycle phases A1–A3, extraction of raw materials, transport, and processing, are analyzed in this study [8]. For the functional unit, a winder staircase with five steps and an angle of 90° was chosen, as in Figure 1.

The case study of a 3D printed lost formwork for a winding staircase illustrates the potential of additive manufacturing to achieve material efficiency and design adaptability in construction.

**Figure 2:**
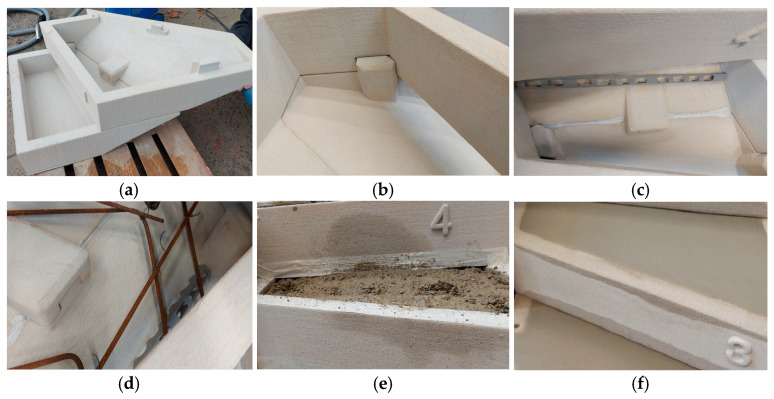
Production process of staircase with additively manufactured lost formwork: (**a**) assembly of individually printed concrete throughs; (**b**) stacked throughs held together by joints; (**c**) sealing connections between elements; (**d**) insertion of reinforcement; (**e**) pouring of concrete infill; (**f**) smoothing of step surfaces.

A promising approach to achieving efficiency improvement is the digital fabrication of building components, with a focus often on direct 3D printing with concrete. However, in this study, an alternative approach was pursued, focusing on indirect digital fabrication using a 3D printed integrated formwork. Staircase elements were produced using a 3D concrete printer, stacked, reinforced, and then concreted to achieve full load-bearing capacity (Figure 2). As demonstrated in Figure 2, the additive manufacturing process achieved a high-resolution surface finish, eliminating the need for any additional surface treatment.

A key advantage of the digital construction process employed is its ability to address the current skilled labor shortage in the construction industry. The lack of skilled workers, especially in traditional trades such as formwork construction and concrete processing, poses significant challenges for the industry. The use of 3D printing technologies offers a solution here, as it reduces the labor intensity and complexity of certain construction processes. This makes it possible to involve unskilled or less-qualified workers in the construction process. Since many of the work steps are automated or greatly simplified, the reliance on highly specialized personnel decreases.

The approach of 3D printed integrated formwork, as applied in the case of the trough staircase, offers further advantages compared to traditional formwork methods. The lost formwork was produced with the particle-bed 3D printing method, specifically Selective Cement Activation (SCA), known for its high resolution compared to other concrete AM methods [15]. SCA offers significant advantages, particularly in terms of design freedom, enabling the creation of complex, customized shapes such as overhangs, arches, and free-form structures without the need for costly formwork. With a high resolution and accuracy down to 0.1 mm, this method allows for the production of intricate and detailed components. However, it is limited by the size of the print area, which means that large components must be produced in sections. While design complexity does not affect production time, large-scale printing still requires significant processing time [16]. SCA enables complex geometries to be realized precisely and efficiently, a process that often requires significant effort using conventional techniques. This is evident, for example, in the view of the staircase from above. The surface created by the winding of the staircase is difficult to achieve with traditional formwork methods without significant effort. The staircase was parametrically designed in Rhinoceros and Grasshopper to be easily adjusted to different usage scenarios. The steps had dimensions of 71.00 cm in width and 20.00 cm in height.

Six different scenarios with varying production methods and materials used (Table 1) were investigated and assessed through a Life Cycle Assessment (LCA).

**Figure 3:**
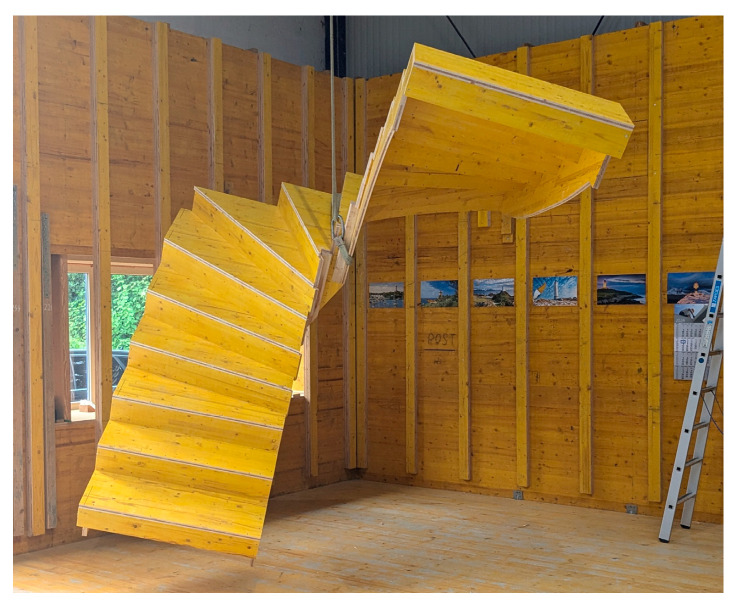
Wooden formwork for a conventional winder staircase.

The first scenario was conventional reinforced concrete production with a one-time use plywood formwork. The second scenario was the conventional manufacturing method as well, but using a one-time use untreated timber formwork. The formwork utilized in scenarios 1 and 2 closely resembled the design depicted in Figure 3.

The choice of single-use formwork was dictated by the unique specifications of the staircase design, which required a tailored approach to meet its complex geometry. The use of a re-usable formwork was deemed unsuitable in this context, as it lacked the flexibility to meet the precise contours and dimensions required for this particular design. Instead, a specifically fabricated formwork was required to ensure that the architectural and functional features of the staircase were accurately realized. Conventional one-time use wood formwork performs best in terms of environmental impact compared to plastic or steel formwork [11,12]. Therefore, the materials plywood and timber were chosen to be analyzed.

For Scenarios 3 to 6, a permanent formwork with a thickness of 5 cm was printed by a SCA 3D printer, resulting in a smaller amount of filling with the conventional reinforced concrete mixture used in Scenarios 1 and 2. In Scenario 3 and 5 the formwork was printed with mixtures designed by Herding et al. [15]. To address the significant influence of cement dosage on the environmental impact of 3D printed concrete (3DPCC), the scenarios were deliberately designed with a reduced cement content. This approach considered the cement requirements necessary to achieve the printing properties, which were notably higher compared to conventional cast concrete [18]. These mixtures were changed for Scenarios 4 and 6 by replacing the Portland cement of the printed formwork with CEM III/B, a ground granulated blast furnace slag cement (GGBFS). Clinker substitutes such as GGBFS cements play a critical role in reducing greenhouse gas emissions in cement production by lowering the clinker content, which is the main source of emissions [19]. GGBFS, a by-product of the steel industry, exhibits hydraulic properties when mixed with clinker and gypsum, enabling its use as a partial replacement for clinker. Although these substitutes reduce process-, fuel-, and power-related CO_2_-equivalent emissions, they may require additional energy for fine grinding, potentially offsetting gains in energy efficiency [19,20]. Despite its current limitations, such as not being printable for SCA, GGBFS cement was chosen for its high mitigation potential and role in optimizing resource use. While not yet applied to SCA, this study provides a foundation to encourage further exploration of GGBFS cement for future advancements.

### 2.2. Life Cycle Inventory (LCI)

The scenarios already described in [17] take into account both the input and output processes (materials and produced waste). The machines used in the processes and the electricity were not included due to lack of data on particle-bed 3D printers used in construction. To maintain comparability, the machines and electricity were therefore not included in the conventional scenarios either.

The datasets utilized in this LCA were exclusively sourced from the ecoinvent3.9.1 database, which was released on the 15th of December in 2022. To evaluate the impacts of stages A1 to A3, market datasets from ecoinvent3.9.1 were employed.

The selection of geographical locations followed a hierarchical approach: priority was given to datasets representing Europe as a whole. If no such dataset was available, datasets for “Europe without Switzerland” were used. In cases where neither of these options was applicable, datasets from specific European countries were selected. If no suitable European dataset was available, the global dataset was used as a last resort.

Scenarios 1 and 2 comprised two components: a one-time use wooden formwork and the corresponding concrete filling for the staircase. Scenarios 3 to 6 utilized a SCA 3D printed lost formwork in combination with the concrete filling.

The concrete used for the filling was a generic mixture of Ordinary Portland cement with an approximate density of 2400 kg/m^3^. This concrete composition remained consistent across all six scenarios.

In Scenarios 1 and 2, the quantity of wood required for the one-time use formwork of the staircase was determined based on the surface area of the functional unit [21]. The nails needed for the formwork were not considered. The waste concrete was measured as 60 kg of unused material, which was disposed of. The waste wood was the one-time used wood formwork as the staircase was a non-standardized one and was only produced once. The total mass of the waste wood was calculated based on the density of the specific wood type used. Additionally, the wastewater from the concrete production was the water used to clean the equipment after the concrete work was finished. The LCI of Scenario 1 is seen in Table 2. For Scenario 2, the plywood formwork was replaced by timber using the dataset “market for cleft timber, measured as dry mass|cleft timber, measured as dry mass|Cutoff, U—Europe without Switzerland”.

In Scenarios 3 to 6, the additively manufactured scenarios, the volume of concrete filling required for the staircase was reduced by 40% compared to Scenarios 1 and 2. This reduction was attributed to the presence of the SCA-printed lost formwork, which occupied a portion of the total volume, thereby decreasing the overall concrete demand. Scenario 3 utilized a mixture composition of 60/0/40_0.5, as described by Herding et al. [15]. The Life Cycle Inventory (LCI) for Scenario 5, calculated using the 80/0/20_0.5 mixture composition by Herding et al. [15], is presented in Table 3. In Scenarios 4 and 6, the Portland cement was replaced by CEM III/B “market for cement, CEM III/B|cement, CEM III/B|Cutoff, U—Europe without Switzerland”.

Even though the mixture was not yet printable, it was modeled to show the environmental potential of low-clinker cementitious mixtures. The waste concrete was measured to be 36 kg, reflecting the overall reduction in the concrete filling compared to Scenarios 1 and 2. The same equipment was used for producing the concrete filling as the concrete in Scenarios 1 and 2, and, therefore, the same amount of wastewater was required for cleaning it. The LCI of Scenario 5 is seen in Table 3.

### 2.3. Life Cycle Impact Assessment (LCIA)

The impact categories Global Warming Potential (GWP); Ozone Depletion Potential (ODP); Acidification Potential (AP); eutrophication potential—aquatic freshwater (EP-freshwater); eutrophication potential—aquatic marine (EP-marine); eutrophication potential—terrestrial (EP-terrestrial); abiotic depletion potential for non-fossil resources (ADPE); abiotic potential for fossil resources (ADPF); water use (WDP); total use of renewable primary energy resources (PERT); and total use of non-renewable primary energy resources (PENRT) were determined according to DIN EN 15804 [8]. The results of ADPE, ADPF, and WDP should be used with care as the uncertainties of the results are high and as there is limited experience with the indicator.

The selection of impact categories in this study aligns with the core environmental indicators specified in DIN EN 15804 [8], reflecting their relevance and importance in LCAs. Global Warming Potential (GWP) and Ozone Depletion Potential (ODP) are classified as Type 1 impact categories, indicating that they are recommended and considered satisfactory due to their high environmental significance and widespread acceptance within both scientific and regulatory frameworks [8,22]. The alignment of the GWP and ODP categories with policy, their scientific credibility, and their adaptability to varying emission profiles make them a key tool for evaluating climate impacts in LCAs [22]. The Intergovernmental Panel on Climate Change (IPCC)’s 100-year timeframe model for calculating GWP and the World Meteorological Organization’s (WMO)’s model for calculating ODP are the most recent and reliable midpoint models available for these categories [8,23]. Both indicators are fundamental to capturing critical aspects of environmental impact, as outlined in the standard.

Acidification Potential (AP); eutrophication potential—aquatic freshwater (EP-freshwater); eutrophication potential—aquatic marine (EP-marine); and eutrophication potential—terrestrial (EP-terrestrial) are classified as Type 2 categories, meaning they are also recommended, though some methodological improvements or refinements are necessary to enhance their robustness and applicability [8,22]. Acidification Potential (AP) and terrestrial eutrophication potential (EP-terrestrial) are calculated with the Accumulated Exceedance method and are highly applicable on a European scale, with strong environmental relevance for biodiversity and the natural environment. They provide a comprehensive assessment of atmospheric and soil impacts. However, they are sensitive to the emission scenario and current critical load, which may affect their reliability under varying conditions [8,22,23]. The aquatic eutrophication potentials (EP-freshwater and EP-marine), calculated with the recommended midpoint ReCiPe method, effectively assess aquatic ecosystem impacts with high environmental relevance. However, they do not cover terrestrial ecosystems and are parameterized for the US with spatial differentiation only at the state level, limiting their comprehensiveness. In summary, while these Type 2 categories are valuable, improvements in scope, spatial differentiation, and comprehensiveness are needed to enhance their scientific applicability.

Lastly, abiotic depletion potential for non-fossil resources (ADPE), abiotic potential for fossil resources (ADPF), and water use (WDP), are categorized as Type 3 indicators, suggesting that while they are part of the core set of indicators under DIN EN 15804, their application requires caution due to uncertainties or limitations in their assessment methodologies [10,22]. The CML method for finding the abiotic depletion potentials (ADPE and ADPF) is the recommended one, as it considers the extraction and the reserves of a resource. To assess WDP, the recently developed AWARE method was used. Due to the young age of the method, it lacks significance and needs further expertise and experience [8,24].

The uncertainties associated with the choice of the total use of renewable primary energy resources (PERT) and total use of non-renewable primary energy resources (PENRT) cumulative energy demand (CED) model is relatively low. The analysis of cumulative energy demand is valuable for obtaining a general overview of the energy-related environmental impacts throughout a product’s life cycle and serves as an initial comparative tool for assessing individual products [25].

While Type 1 categories such as GWP and ODP are well-established and critical for Life Cycle Assessments, Type 2 categories like AP and EP offer valuable insights into other environmental impacts but require further refinement to improve their robustness and applicability. The Type 3 indicators provide useful data on resource depletion and water use but should be applied with caution due to uncertainties in their methodologies. The parameters for describing the use of resources (PERT and PENRT) are beneficial as they provide a comprehensive assessment of energy demand, helping to identify and compare energy-related environmental impacts across different product systems. Overall, continued advancements in the methodologies and broader scope of these indicators will enhance their effectiveness in supporting comprehensive Life Cycle Assessments, offering more accurate and reliable assessments of environmental sustainability.

## 3. Results and Interpretation

### 3.1. Results

In Section 2.1, during the selection of the scenarios, an optimization was already considered through the use of CEM III, the GGBFS cement. Additionally, the calculation of concrete volume and the wood consumption for the one-time use formwork were adjusted according to the specific scenarios. Consequently, the amount of waste was also adapted to each respective scenario.

Table 4 presents the results of the six scenarios, providing direct value comparison for each impact category. The analysis shows that the highest Global Warming Potential is observed in Scenario 1, with 276 kg CO_2_ eq., followed closely by Scenario 3 at 246 kg CO_2_ eq., underscoring the significant environmental impact of plywood. Moreover, altering the additive manufacturing mixture ratio from 60/40 to 80/20 results in a notable reduction of approximately 29% in CO_2_ emissions, as evidenced by the comparison between Scenarios 3 and 5. The utilization of CEM III in Scenario 4 accounts for a reduction of 33% in CO_2_ emissions compared to Scenario 3. Scenario 6 falls below 50% of the values of Scenario 1 in every single impact category. The biggest difference between these scenarios is for the PERT, where Scenario 6 only has 0.7% of the MJ eq. of Scenario 1. Considering combined primary energy resources (PEs), Scenario 6 consumes 7% MJ eq. in comparison to Scenario 1 due to the higher PENRT levels. The data indicates that the transition from Ordinary Portland cement to GGBFS cement is responsible for the observed increase in ADPE and PERT. The increased use of renewable primary energy resources is counterbalanced by a more significant reduction in non-renewable energy use. Consequently, although PERT increases, the overall primary energy demand decreases when CEM III/B is used.

### 3.2. Interpretation and Dominance Analysis

The staircase serves as a conceptual framework that can take various forms, allowing for flexibility in its application. Rather than focusing on absolute data, the emphasis is on percentage-based results that highlight the potential impact and scalability of the approach. This perspective provides a more insightful understanding of relative gains, enabling users to appreciate how incremental improvements can add up. By concentrating on percentage outcomes, we can effectively illustrate the broader possibilities and adaptability of the concept without being confined to fixed data points or rigid structures.

**Figure 4:**
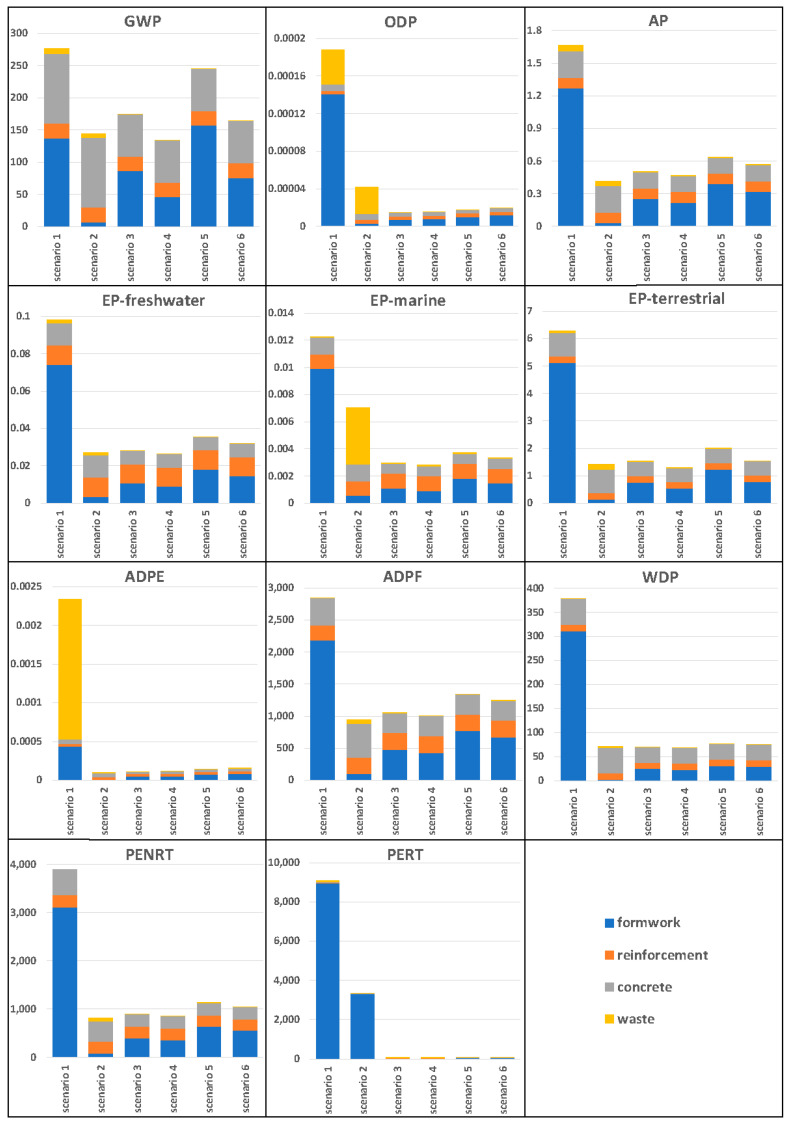
Results of Scenario 1 (conventional production with plywood formwork), Scenario 2 (conventional production with timber formwork), Scenario 3 (additively manufactured formwork with 60/40 mixture [15]), Scenario 4 (additively manufactured formwork with 60/40 mixture [15] and CEM III), Scenario 5 (additively manufactured formwork with 80/20 mixture [15]), and Scenario 6 (additively manufactured formwork with 80/20 mixture [15] and CEM III).

Figure 4 depicts the overall results of every scenario. It is visible that Scenario 1 has the highest environmental impact in every category. The comparison of Scenarios 1 and 2 supports the findings that the change from plywood to timber is a severe improvement on the sustainability of the staircase. Balasbaneh et al. [11] determined that the GWP of single-use plywood formwork per square meter exceeds that of timber, a finding reaffirmed by the results of this study. Scenario 3, compared to Scenario 5, highlights the impact of a lower cement content for the permanent formwork. Scenarios 3 to 6 show that the substitution of the Portland cement with GGBFS cement lowers every category except for ODP, ADPE, and PERT, where the values are only negligibly higher than the original mixture compositions. Therefore, it can be stated that Scenario 6 performs comparably best and has the lowest values of GWP, EP-freshwater, EP-marine, EP-terrestrial, and WDP, and the second-lowest in the other categories except for ADPE.

Figure 4 also illustrates the division of the four main components of the staircase and their influence on each impact category. The change in dominance between Scenarios 1 and 2 is based on the change from plywood to timber formwork. Scenarios 3 to 6 have a certain similarity as the reinforcement, concrete, and waste are unchanged, and only the formwork with the different mixtures are altered. The waste is only a minor contributor for the 3D printed scenarios, and an increased one for the conventional productions, especially for ODP.

**Figure 5:**
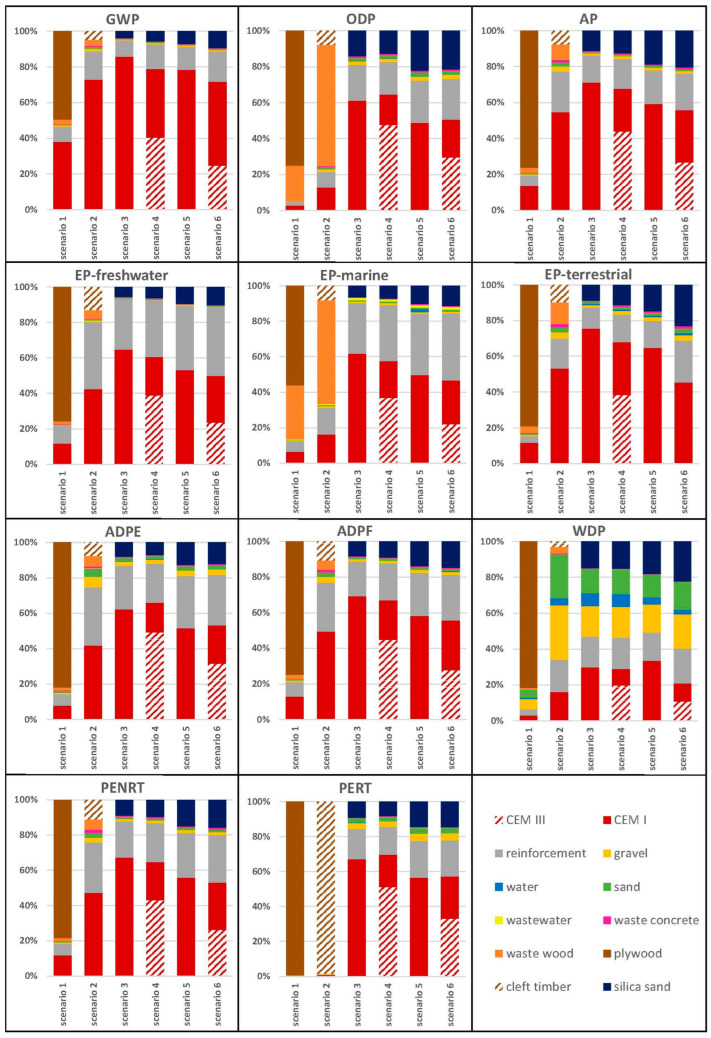
Analysis of the six scenarios. Dominance analysis separated by material.

Figure 5 presents that the main contributor to each category of Scenario 1 is the plywood formwork, whose percentage share stays at almost 80% for every impact category except for GWP, EP-marine, and the PERT. Notably, 99% of the total PERT is attributed to the plywood formwork. The environmental improvement resulting from the shift from plywood to timber formwork is clearly highlighted in the visualization. Figure 5 highlights that cement is the highest contributor in all the scenarios, except when plywood is a part of them. The amount of reinforcement used in the scenarios is consistent across all cases. In conventional scenarios, waste plays a more significant role compared to additive manufacturing scenarios. The staircase built using the additive-manufactured optimized mixture and the low-clinker cement formwork outperformed the optimal single-use timber formwork (Scenario 2) across various environmental categories.

## 4. Discussion

### 4.1. Cement Datasets

Concrete is the most used material in the construction industry, and it is estimated that the production of cement, its main binder, is responsible for approximately 7% of global carbon emissions [26]. Additionally, the location of cement production plays a critical role in determining the total emissions. Table 5 and Table 6 highlight the geographical influence on GWP of the cement datasets. Across all regions analyzed, the vast majority of the GWP from cement production—ranging from 95.5% to 99.5%—originates from cement production, with transportation accounting for the remaining emissions. Switzerland has the highest share of emissions from cement production (99.5%), while India shows the lowest (95.5%), indicating that the transportation factor for Switzerland is very low compared to other regions. Other regions, including South Africa, Peru, the US, Europe, and Canada, report similar proportions, with cement production contributing approximately 97%.

For the CEMIII/B dataset, there are only three geographical locations in the ecoinvent3.9.1 database available. For the “Rest of World” category, 93.6% of emissions are from cement production, while Europe without Switzerland shows a slightly higher share at 94.0%. Switzerland has the highest proportion, with 98.7% of emissions from cement production, leaving only a small fraction attributable to transportation.

The results indicate that factors such as the energy mix of the region (e.g., reliance on coal, natural gas, or renewable energy), transportation distances for raw materials, and the efficiency of local production technologies can significantly influence the carbon footprint of cement production.

The data in Table 5 and Table 6 clearly illustrate that CEM III/B achieves more than a 50% reduction in GWP compared to Portland cement in the same geographical location. This demonstrates the effectiveness of CEM III/B in mitigating environmental impacts, making it a more sustainable alternative to traditional Portland cement.

Further reductions in conventional production scenarios can be achieved by replacing Portland cement with GGBFS cement in the concrete mixture, a strategy that also applies to the filling of the additive-manufactured lost formwork.

Reducing the clinker-to-cement ratio is a key strategy for lowering the GWP in cement production, but it is not the only solution. Electrifying production processes with renewable energy sources is another, and the scaling up of emerging technologies and alternative binders offer promising pathways for decarbonization [26]. A novel approach involves using captured CO_2_ to produce nano-CaCO_3_ directly within the plant, which is then mixed with cement clinkers to create a new generation of cement. This method can reduce CO_2_ emissions by up to 70% (from 0.96 kg to 0.3 kg CO_2_ eq.) while improving durability, extending the lifespan of structures, and lowering the overall environmental impact of cement production and construction [27,28].

In this study, ecoinvent3.9.1 was used as it was the most current and validated version available at the time the study began. During the research, the evoinvent3.10 database was released. Table 7 and Table 8 show comparisons of the cement datasets used in the study, the updated one, and their deviation.

The values for Portland cement for the impact categories ADPE and PERT increase by 140.67% and 15.83%, respectively. While the PERT rises in evoincent3.10, the PENRT falls by 1.41%, which is positive overall as the primary energy has decreased and, within it, the amount of renewable energy has increased. For the GGBFS cement, the values in ecoinvent3.10 have increased for the impact categories GWP, AP, ADPE, and PERT. These results suggest an evolving understanding of the life cycle impacts of cement production, with potential shifts in energy sourcing, modeling assumptions, or updated input data in the ecoinvent3.10 database.

Table 9 and Table 10 present a comparison of Portland cement and GGBFS cement data from the ecoinvent3.10 and oekobaudat databases, highlighting significant deviations across various environmental impact categories. The deviations between the databases range from 18% (GWP) to 149% (PERT), indicating substantial discrepancies in how environmental impact is assessed. These discrepancies are influenced not only by variations in data interpretation but also by the geographic scope of the databases: ecoinvent refers to Europe without Switzerland, while oekobaudat is a database that focuses specifically on Germany. Such large deviations emphasize the importance of carefully selecting and cross-referencing databases when conducting environmental impact assessments.

Further research into advanced low-carbon binders, such as Celitement, could contribute significantly to reducing greenhouse gas emissions from cement. Celitement is an environmentally advantageous hydraulic binder that, when used as a replacement for Portland cement clinker, can reduce CO_2_ emissions by up to 50% during production, making it a more sustainable alternative in cement manufacturing [29].

As the cement industry transitions towards more sustainable practices, the role of robotics and electricity consumption in production is expected to become increasingly influential in the overall environmental impact assessment. Moreover, thorough research into electricity consumption and its impact on the Life Cycle Assessment (LCA) of additive manufacturing products in construction will become increasingly essential. Understanding the energy demands and environmental implications of using electricity in these processes will be crucial for evaluating the overall sustainability and decarbonization potential of additive manufacturing technologies in the construction sector.

### 4.2. Energy Datasets

Considering the significance of electricity locations, it is therefore crucial to analyze the electricity mix used in the case of the SCA-printed lost formwork staircase. This study identifies plywood, Ordinary Portland cement, and GGBFS cement as the three largest contributors to GWP. On closer examination of the dataset “market for plywood|plywood|Cutoff, U—RER”, 38.32% of the GWP is caused by the electricity used in the production process for the plywood. For plywood production, the electricity used in the market process in ecoinvent3.10 is medium voltage in Europe, while for both types of cement, the electricity mix is based on medium voltage in Europe without Switzerland. As observed in Table 11, there are significant variations in carbon dioxide emissions across different regions. These values show that Switzerland and France have notably lower carbon intensities due to their reliance on low-carbon energy resources (such as nuclear or hydroelectric power), while countries like China and Germany have higher carbon intensities due to a greater reliance on coal and other fossil fuels. Considering the values presented in Table 11, the overall sustainability of the SCA staircase is influenced by the electricity mix of the location.

While the cement industry holds significant potential for reducing its carbon footprint through various strategies, another critical factor in reducing the environmental impact is the energy mix used in the production processes of the product. The environmental impact of innovative technologies like robotic 3D printing in construction is closely tied to the energy sources that power these processes. As the cement industry continues to lower its Global Warming Potential, the relative impact of robotic processes and electricity consumption will become more significant in the overall environmental footprint of construction.

Kuzmenko [30] found that the environmental impact of the robotic 3D printing process is significantly influenced by both its lifespan and the local electricity mix, with the overall process-related impact being roughly evenly split between embodied and operational emissions. The supplementary burden associated with the six-axis extrusion robotic printing technology contributes approximately 13% to the overall climate change impact. Additionally, factors like printing resolution further influence the overall environmental footprint, particularly the GWP [30]. In contrast, direct emissions from conventional onsite construction contribute only 2.42% to the overall climate change impact [31]. The Environmental Product Declarations (EPDs) from Informationszentrum Beton GmbH indicate that the production processes of C12/15 and C20/25 concrete contribute approximately 3.0% to 4.0% of the total GWP in the A1–A3 life cycle stages [32]. These compressive strength categories closely resemble the compressive strength of the additive-manufactured mixtures in Scenario 3 and Scenario 5 [15]. Conventional construction methods exhibit a lower process-related GWP, contributing approximately 2.42–4.00% to the overall climate change impact, whereas additive manufacturing processes impose a higher environmental burden. However, these percentages of the different production processes can vary depending on regional energy sources, as shown in Table 11, with some regions experiencing a considerably higher process-related impact.

Overall, data on the impact of robotics and electricity consumption in additive manufacturing for construction remains very limited. Kuzmenko has conducted a thorough study on extrusion-based 3D printing, providing valuable insights into its environmental impact. However, the availability of data on particle-bed 3D printing, particularly regarding energy consumption, remains limited, constraining the scope of this study, which utilizes a particle-bed 3D printer. The GWP associated with the robotic printing process is notably high and can significantly influence the overall environmental impact of the 3D printing process. However, further investigations are necessary to determine whether the particle-bed printing process exhibits a similar level of impact as the extrusion-based process.

## 5. Conclusions and Outlook

This paper examines strategies to reduce the environmental impact of a 3D printed lost formwork for a winding staircase, highlighting the potential of additive manufacturing to enhance material efficiency and design flexibility in construction. Among the six analyzed scenarios, the additive manufacturing scenario utilizing an already optimized 60/40 mixture [15] for the lost formwork of the staircase demonstrates the best LCA results. In this scenario, the Portland Cement content was substituted with GGBFS cement for the SCA-printed lost formwork for the staircase. However, current mixtures with GGBFS for additive manufacturing processes are not yet printable. This study aims to highlight the significance of such substitutions, providing material researchers with insights into the environmental benefits and encouraging further development of printable, low-clinker cementitious materials. Therefore, further research is needed to lower the clinker content in additive manufacturing cement mixtures without compromising the performance.

A crucial limitation of this study is the exclusion of machinery and equipment, along with their respective electricity consumption, from the LCI. This decision was made due to the current lack of available data on particle-bed 3D printers for construction applications. To maintain comparability, the machines and electricity are therefore not included in the conventional scenarios either. Nevertheless, the environmental impact of robotic 3D printing processes is closely tied to the energy mix used. As such, the sustainability of robotic 3D printing processes cannot be fully evaluated without considering the variability in electricity mixes across different regions.

One of the primary contributions of this research is that it is among the first studies to analyze a particle-bed 3D printed lost formwork using an LCA approach. The findings illustrate both the opportunities and challenges of integrating additive manufacturing into construction. In essence, the example of a 3D printed staircase serves as a microcosm of broader trends shaping the construction industry’s sustainability journey. By leveraging technology, creativity, and collaboration, stakeholders can pave the way for a greener, more sustainable built environment that meets the needs of present and future generations.

Further studies are needed to evaluate the environmental viability of 3D printed formwork based on its complexity. This research lays the groundwork for exploring the circularity potential, life cycle phases (B, C, and D), and cost efficiency of 3D printed lost formwork. Future research should also include robotic systems, their wear components, electricity consumption, and the overall lifespan of different robotic systems. The focus should be not only on cement-based materials, but also on alternative materials such as earth or steel. This research could help improve the sustainability and resource efficiency of additive manufacturing and identify new ways of using environmentally friendly materials in the construction industry. These topics are currently researched within the TRR277 [33], contributing to a deeper understanding of the sustainability and circularity of additive manufacturing in construction.

## Figures and Tables

**Table 1:** Six scenarios with varying production methods and materials used, adapted from [17] and licensed by Springer Nature Customer Center GmbH.

Name	Type of Formwork	Material for Formwork
Scenario 1	Conventional one-time use wood formwork	plywood
Scenario 2	Conventional one-time use wood formwork	timber
Scenario 3	SCA 3D printed lost formwork	60/40 volumetric share of aggregate/cement [15] mixture with CEM I
Scenario 4	SCA 3D printed lost formwork	60/40 volumetric share of aggregate/cement [15] mixture with CEM III
Scenario 5	SCA 3D printed lost formwork	80/20 volumetric share of aggregate/cement [15] mixture with CEM I
Scenario 6	SCA 3D printed lost formwork	80/20 volumetric share of aggregate/cement [15] mixture with CEM III

**Table 2:** LCI of Scenario 1—conventional reinforced concrete with plywood formwork, adapted from [17] and licensed by Springer Nature Customer Center GmbH.

Material	Process	Amount	**Unit**
*Concrete filling*			
Cement, Portland	market for cement, Portland|cement, Portland|Cutoff, U—Europe without Switzerland	119.70	kg
Gravel, round	market for gravel, round|gravel, round|Cutoff, U—CH	349.30	kg
Reinforcing steel	market for reinforcing steel|reinforcing steel|Cutoff, U—GLO	10.63	kg
Sand	market for sand|sand|Cutoff, U—CH	285.95	kg
Tap water	market for tap water|tap water|Cutoff, U—Europe without Switzerland	63.00	kg
Waste concrete, not reinforced	market for waste concrete, not reinforced|waste concrete, not reinforced|Cutoff, U—Europe without Switzerland	60.00	kg
Wastewater from concrete production	market for wastewater from concrete production|wastewater from concrete production|Cutoff, U—CH	0.09	m^3^
*One-time use wood formwork*			
Plywood	market for plywood|plywood|Cutoff, U—RER	0.32904	m^3^
Waste wood, untreated	market for waste wood, untreated|waste wood, untreated|Cutoff, U—AT	236.9088	kg

**Table 3:** LCI of Scenario 5—80/20 mixture for SCA 3D printed formwork and reinforced concrete filling, adapted from [17] and licensed by Springer Nature Customer Center GmbH.

Material	Process	Amount	Unit
*Concrete filling*			
Cement, Portland	market for cement, Portland|cement, Portland|Cutoff, U—Europe without Switzerland	71.82	kg
Gravel, round	market for gravel, round|gravel, round|Cutoff, U—CH	209.58	kg
Reinforcing steel	market for reinforcing steel|reinforcing steel|Cutoff, U—GLO	10.63	kg
Sand	market for sand|sand|Cutoff, U—CH	171.57	kg
Tap water	market for tap water|tap water|Cutoff, U—Europe without Switzerland	37.80	kg
Waste concrete, not reinforced	market for waste concrete, not reinforced|waste concrete, not reinforced|Cutoff, U—Europe without Switzerland	36.00	kg
Wastewater from concrete production	market for wastewater from concrete production|wastewater from concrete production|Cutoff, U—CH	0.09	m^3^
*SCA 3D printed lost formwork*			
Cement, Portland	market for cement, Portland|cement, Portland|Cutoff, U—Europe without Switzerland	83.72	kg
Silica sand	market for silica sand|silica sand|Cutoff, U—GLO	296.80	kg
Tap water	market for tap water|tap water|Cutoff, U—Europe without Switzerland	34.86	kg

**Table 4:** Results of six scenarios.

	GWP kg CO_2_ eq.	ODP kg CFC-11 eq.	AP molc H+ eq.	EP-Freshwater kg P eq.	EP-Marine kg N eq.	EP-Terrestrial molc N eq.	ADPE kg Sb eq.	ADPF MJ	WDP m^3^	PENRT MJ eq.	PERT MJ eq.
Scenario 1	276	1.88 × 10^−4^	1.67	0.098	0.018	6.47	4.37 × 10^−4^	2918	382	3979	9013
Scenario 2	144	4.19 × 10^−5^	0.42	0.027	0.007	1.43	1.03 × 10^−4^	944	72	811	3345
Scenario 3	246	1.78 × 10^−5^	0.64	0.035	0.004	2.01	1.38 × 10^−4^	1346	77	1134	80
Scenario 4	164	1.94 × 10^−5^	0.57	0.032	0.003	1.55	1.53 × 10^−4^	1252	75	1053	86
Scenario 5	176	1.52 × 10^−5^	0.51	0.028	0.003	1.57	1.11 × 10^−4^	1074	83	915	66
Scenario 6	134	1.56 × 10^−5^	0.47	0.026	0.003	1.31	1.19 × 10^−4^	1008	68	860	67

**Table 5:** Comparison of 1 kg of Portland cement “market for cement, Portland|cement, Portland|Cutoff, U” from Switzerland, Canada, Europe without Switzerland, United States, Peru, India, and South Africa in ecoinvent3.9.1 database.

Market Process Medium Voltage	Switzerland	Canada	Europe/CH	US	Peru	India	South Africa
GWP [kg CO_2_ eq.]	0.74008	0.86674	0.880346	0.90786	0.91638	0.93117	1.02766

**Table 6:** Comparison of 1 kg of ground granulated blast furnace slag (GGBFS) cement “market for cement, CEM III/B|cement, CEM III/B|Cutoff, U” from Switzerland, Europe without Switzerland, and Rest of World in ecoinvent3.9.1 database.

Market Process Medium Voltage	Switzerland	Europe/CH	Rest of World
GWP [kg CO_2_ eq.]	0.31174	0.39590	0.44263

**Table 7:** A comparison of the impact categories of 1 kg “market for cement, Portland|cement, Portland|Cutoff, U—Europe without Switzerland” in the ecoinvent3.9.1 and ecoinvent3.10 databases and the deviation.

	GWP kg CO_2_ eq.	ODP kg CFC-11 eq.	AP molc H+ eq.	ADPE kg Sb eq.	ADPF MJ	WDP m^3^	PENRT MJ eq.	PERT MJ eq.
CEM I 3.9.1	0.880346	4.54 × 10^−8^	0.001899	3.59 × 10^−7^	3.191437	0.095574	3.906352	0.223288
CEM I 3.10	0.879083	4.42 × 10^−8^	0.001875	8.64 × 10^−7^	3.164876	0.087237	3.851083	0.258631
Deviation [%]	−0.14	−2.64	−1.26	+140.67	−0.83	−8.72	−1.41	+15.83

**Table 8:** A comparison of the impact categories of 1 kg “market for cement, CEM III/B|cement, CEM III/B|Cutoff, U—Europe without Switzerland” in the ecoinvent3.9.1 and ecoinvent3.10 databases and the deviation.

	GWP kg CO_2_ eq.	ODP kg CFC-11 eq.	AP molc H+ eq.	ADPE kg Sb eq.	ADPF MJ	WDP m^3^	PENRT MJ eq.	PERT MJ eq.
CEM III/B 3.9.1	0.396287	5.53 × 10^−8^	0.001495	4.51 × 10^−7^	2.706048	0.089025	3.343201	0.262794
CEM III/B 3.10	0.396783	5.20 × 10^−8^	0.001514	1.11× 10^−6^	2.532155	0.059845	3.098149	0.276835
Deviation [%]	+0.13	−5.97	+1.27	+146.12	−6.43	−32.78	−7.33	+5.34

**Table 9:** A comparison of the impact categories of 1 kg “market for cement, Portland|cement, Portland|Cutoff, U—Europe without Switzerland” in the ecoinvent3.10 database and “Portland cement (CEM I)” phase A1–A3 in the oekobaudat database and the deviation.

	GWP kg CO_2_ eq.	ODP kg CFC-11 eq.	AP molc H+ eq.	ADPE kg Sb eq.	ADPF MJ	WDP m^3^	PENRT MJ eq.	PERT MJ eq.
CEM I 3.10	0.879083	4.42 × 10^−8^	0.001875	8.64 × 10^−7^	3.164876	0.087237	3.851083	0.258631
CEM I oekobaudat	0.665000	2.11 × 10^−11^	0.000940	4.91 × 10^−8^	2.060000	0.004690	2.060000	0.644000
Deviation [%]	−24.35	−99.95	−49.87	−94.32	−34.91	−94.62	−46.51	+149.00

**Table 10:** A comparison of the impact categories of 1 kg “market for cement, CEM III/B|cement, CEM III/B|Cutoff, U—Europe without Switzerland” in the ecoinvent3.10 database and “Blastfurnace cement CEM III/B 42.5 L-LH/SR” phase A1–A3 in the oekobaudat database.

	GWP kg CO_2_ eq.	ODP kg CFC-11 eq.	AP molc H+ eq.	ADPE kg Sb eq.	ADPF MJ	WDP m^3^	PENRT MJ eq.	PERT MJ eq.
CEM III/B 3.10	0.396783	5.20 × 10^−8^	0.001514	1.11 × 10^−6^	2.532155	0.059845	3.098149	0.276835
CEM III/B oekobaudat	0.327000	7.10 × 10^−12^	0.000520	3.85 × 10^−8^	1.410000	0.007850	1.410000	0.519000
Deviation [%]	−17.59	−99.99	−65.65	−96.53	−44.32	−86.88	−54.49	+87.48

**Table 11:** kg CO_2_ eq. per kWh of electricity, medium voltage of different locations assessed with ecoinvent3.10.

Market Process Medium Voltage	Europe	Switzerland	Germany	France	China	Canada
GWP [kg CO_2_ eq.]	0.32536	0.02332	0.42080	0.07711	0.95041	0.18963

## Data Availability

The original contributions presented in this study are included in the article. Further inquiries can be directed to the corresponding author.

## Abbreviations

The following abbreviations are used in this manuscript:

3DPCC	3D printed concrete
AP	Acidification Potential
CED	cumulative energy demand
E-LCA	Environmental Life Cycle Assessment
EPD	Environmental Product Declaration
EP-freshwater	eutrophication potential—aquatic freshwater
EP-marine	eutrophication potential—aquatic marine
EP-terrestrial	eutrophication potential—terrestrial
GGBFS	ground granulated blast furnace slag
GWP	Global Warming Potential
IPCC	Intergovernmental Panel on Climate Change
LCA	Life Cycle Assessment
LCI	Life Cycle Inventory
LCIA	Life Cycle Impact Assessment
ODP	Ozone Depletion Potential
PE	primary energy resources
PENRT	total use of non-renewable primary energy resources
PERT	total use of renewable primary energy resources
POCP	Photochemical Ozone Creation Potential
SCA	Selective Cement Activation
WMO	World Meteorological Organization
